# The TKI Era in Chronic Leukemias

**DOI:** 10.3390/pharmaceutics13122201

**Published:** 2021-12-20

**Authors:** Danilo De Novellis, Fabiana Cacace, Valeria Caprioli, William G. Wierda, Kris M. Mahadeo, Francesco Paolo Tambaro

**Affiliations:** 1 Hematology and Transplant Center, University “Hospital San Giovanni di Dio e Ruggi D’Aragona”, 84131 Salerno, Italy; 2 Unità Operativa di Trapianto di Cellule Staminali Ematopoietiche e Terapie Cellulari, Azienda Ospedaliera di Rilievo Nazionale Santobono-Pausilipon, 80123 Napoli, Italy; fabianacacace@santobonopausilipon.it (F.C.); valeriacaprioli@santobonopausilipon.it (V.C.); francescopaolotambaro@santobonopausilipon.it (F.P.T.); 3 Department of Leukemia, The University of Texas MD Anderson Cancer Center, Houston, TX 77030, USA; wwierda@mdanderson.org; 4 Pediatric Stem Cell Transplantation and Cellular Therapy, CARTOX Program, University of Texas at MD Anderson Cancer Center, Houston, TX 77030, USA; KMMahadeo@mdanderson.org

**Keywords:** chronic myeloid leukemia (CML), chronic lymphocytic leukemia (CLL), BCR-ABL1-inhibitors, BTK-inhibitors, PI3K-inhibitors, targeted therapy, treatment discontinuation

## Abstract

Tyrosine kinases are proteins involved in physiological cell functions including proliferation, differentiation, and survival. However, the dysregulation of tyrosine kinase pathways occurs in malignancy, including hematological leukemias such as chronic myeloid leukemia (CML) and chronic lymphocytic leukemia (CLL). Particularly, the fusion oncoprotein BCR-ABL1 in CML and the B-cell receptor (BCR) signaling pathway in CLL are critical for leukemogenesis. Therapeutic management of these two hematological conditions was fundamentally changed in recent years, making the role of conventional chemotherapy nearly obsolete. The first, second, and third generation inhibitors (imatinib, dasatinib, nilotinib, bosutinib, and ponatinib) of BCR-ABL1 and the allosteric inhibitor asciminib showed deep genetic and molecular remission rates in CML, leading to the evaluation of treatment discontinuation in prospective trials. The irreversible BTK inhibitors (ibrutinib, acalabrutinib, zanubrutinib, tirabrutinib, and spebrutinib) covalently bind to the C481 amino acid of BTK. The reversible BTK inhibitor pirtobrutinib has a different binding site, overcoming resistance associated with mutations at C481. The PI3K inhibitors (idelalisib and duvelisib) are also effective in CLL but are currently less used because of their toxicity profiles. These tyrosine kinase inhibitors are well-tolerated, do have some associated in-class side effects that are manageable, and have remarkably improved outcomes for patients with hematologic malignancies.

## 1. Introduction

Tyrosine kinases (TKs) are a large family of surface and intracellular signaling proteins largely conserved in multicellular organisms. Their function is in catalyzing the transfer of a phosphate group from adenosine triphosphate (ATP) to the hydroxyl group of tyrosine residues in specific substrate proteins, creating a strong covalent bond and resulting in energy transfer and signal transduction. The phosphorylation of TKs modulates their activity, promoting the recruitment of multiple downstream signal proteins. Some members are capable of autophosphorylation. This kinase family may be subclassified into transmembrane receptors and non-receptor intracellular signaling TKs [1].

Receptor TKs are transmembrane glycoproteins that physiologically interact with extracellular ligands (often growth factors), transducing the signal from the extracellular environment into the cytoplasm, promoting the dimerization of the receptor first and the autophosphorylation of tyrosine residues [2]. Many cell receptors belong to the TK family and are named according to their ligands (epidermal growth factor receptor (EGFR), fibroblast growth factor receptor (FGFR), nerve growth factor receptor (NGFR)), platelet-derived growth factor receptor (PDGFR), and vascular endothelial growth factor receptor (VEGFR).

Non-receptor TKs is a group of intracellular kinases that are structurally diverse and include Src, Abl, Jak, Ras, and Raf proteins that have similar activities as receptor TKs [1]. Activation of the non-receptor TKs involves aggregation and interactions among intracellular proteins and is characterized by targeted transphosphorylation [3]. Several cell functions commonly associated with cancer, such as proliferation, differentiation, survival, and metabolic impulses are mediated by TK pathways [4].

Chronic myeloid leukemia (CML) [5] and chronic lymphocytic leukemia (CLL) [6], the most common hematological malignancies in the U.S.A and Europe [7], are characterized by a dependence on kinase pathways (see below) for leukemia cell growth and survival, and as a result have been very successfully treated with TK inhibitors. Critical TKs include the fusion chimera oncoprotein BCR-ABL1 in CML [8] and the B-cell receptor (BCR) signaling pathway including Bruton Tyrosine Kinase (BTK) [9] and phosphatidylinositol 3-kinase (PI3K) in CLL [10]. The inhibition of TKs represents the prototype of targeted therapy and has proven to be a key and effective element in the treatment of these hematologic malignancies over the last twenty years. Here, we analyze and describe the role of TKs in the pathophysiology of CML and CLL, and the current tyrosine kinase inhibitor (TKi)-based treatments, both in current practice and clinical trials.

## 2. Chronic Myeloid Leukemia

### 2.1. Kinase Pathways in CML

CML is a clonal myeloproliferative disorder characterized by the Philadelphia (PH) chromosome, which is a genetic alteration resulting from a reciprocal translocation between chromosomes 9 and 22 [t(9;22)] [11]. The incidence rate of CML ranges from 0.6 to two cases per 100,000 individuals [6]. The PH chromosome produces the fusion oncoprotein, BCR-ABL1, which is a constitutively activated kinase responsible for the pathogenesis of CML. Three different isoforms of BCR-ABL1 (p210, p190, and p230) have been described in hematologic malignancies, which are associated with different lengths of the BCR-ABL1 fusion gene. The most common isoform in CML is p210, which is expressed by about 95% of CML patients; the p190 isoform occurs alone in 1–2% and is co-expressed with p210 in 5–7% of patients. The p230 transcript is rare [12,13].

Under physiological conditions, ABL1 is expressed during hematopoietic stem cell development; when fused with BCR, the BCR-ABL1 gene produces a constitutively active protein that loses the ability to migrate into the nucleus and is retained in the cytoplasm where it interacts with several kinases involved in leukemogenesis [14]. The different interactions of BCR-ABL1 with several pathways can be responsible for the process of leukemogenesis. Janus kinase (JAK) and Signal Transducers and Activators of Transcription (STAT) 1, 3, 5, and 6 proteins [15] have been associated with leukemogenesis in PH-positive diseases, leading to the terminal overexpression of oncogene *c-MYC*, which is essential for neoplastic transformation [16].

**Figure 1:**
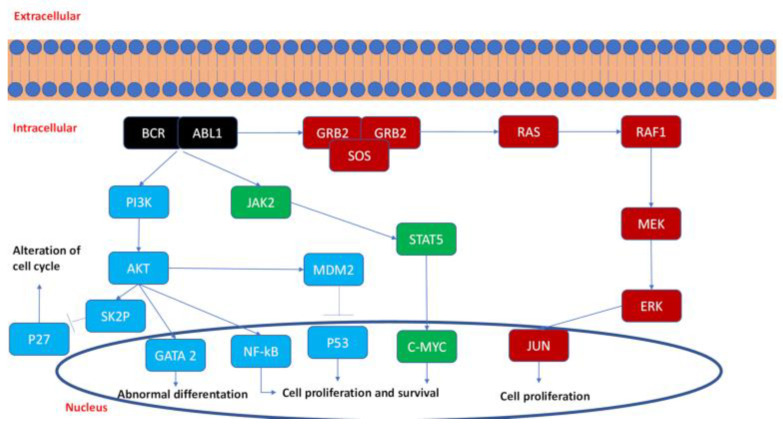
BCR-ABL1 signaling pathway.

In addition, the PI3K–AKT–NF-kB–MM9 pathway is involved in CML pathogenesis. It was shown that the upregulation of this pathway, triggered by the TGF–βinBCR–ABL1 positive hemangioblasts, leads to an enhanced secretion of soluble Kit ligand and ICAM1, promoting the activation and proliferation of CML stem cells [17,18]. Furthermore, PI3K is also responsible for the up-regulation of Skp2, which promotes the degradation of p27, by ubiquitination, and the consequent alteration of cycle cell regulation in CML cells [19]. Interestingly, the inhibition of PI3K-associated pathways prevents BCR-ABL1 leukemogenesis in mice, demonstrating the important role for this kinase in oncogenesis [20]. In addition, the Ras–Raf–MEK–ERK pathway, referred to as the Mitogen-Activated Protein (MAP) kinase pathway, represents one of the most ubiquitous, important, and preserved signaling pathways in multicellular organisms. After the interaction with BCR-ABL1, constitutive Ras activation is triggered, inducing the sequential phosphorylation of multiple Raf–Erk–Mek kinases, whose role is the recruitment of several nuclear transcription factors involved in proliferation [21]. A summary of the BCR-ABL1 pathway is shown in Figure 1.

The fusion oncoprotein BCR-ABL1 and downstream signaling proteins induce pro-survival, proliferation, migration and apoptosis signals in the CML cells.

Blue indicates the PI3K pathway; green is the JAK2 pathway; red is the MAP kinase pathway.

### 2.2. TK Inhibition in CML

The typical CML clinical course consists of three different phases as described by the World Health Organization (WHO) 2016 classification [22]: chronic, accelerated, and blastic phases. Most patients are diagnosed in the chronic phase, which is characterized by <10% blast cells, and no criteria of accelerated phase (at least one of persisting or increasing white blood cell count or splenomegaly unresponsive to therapy, peripheral basophils > 20%, blast cells: 10–19%, platelets count >1000 × 10^9^/L uncontrolled by therapy or <100,000 × 10^9^/L unrelated to therapy, additional clonal chromosomal abnormalities). The blast phase is defined by blast cells >20% or extramedullary involvement.

Historically, treatments for CML consisted of cytoreductive drugs, such as hydroxyurea [23] or busulfan [24]. This approach reduced and controlled peripheral white blood cell count, splenomegaly, and disease-related symptoms but was ineffective in eradicating PH^+^ clones. Therefore, the overall survival (OS) and risk for disease progression were not improved. Interferon-alfa2a was shown to inhibit myeloid cell proliferation [25] and then was compared to conventional cytotoxic chemotherapy (busulfan or hydroxyurea) in a clinical trial. Interferon-alfa2a showed a higher rate of karyotypic responses (defined as >33% of metaphases negative for PH chromosome; 30% vs. 5%), longer median progression-free survival (PFS) (>72 vs. 45 months), and longer median OS (72 vs. 52 months) compared to cytotoxic therapy [26].

Clinical responses and outcomes for patients with CML were remarkably improved after the introduction of a small molecule TK inhibitor of BCR-ABL1, which effectively blocks all the hyperactivated pathways and results in CML cell death [27,28]. The first report of the small molecule inhibitor CGP57-148B was published in 1996; the proliferation rate of the granulocyte–macrophage colony-forming unit (GM-CFU) was potently decreased after exposure to the drug [29]. However, clinical testing and the development of TKi began when the compound CGP57-148B, renamed STI571 and imatinib, was evaluated in a dose-escalating phase I/II trial, which established the maximum tolerated dose as the primary end point and clinical efficacy as the secondary end point. Eighty-three patients with chronic phase CML who failed treatment with interferon received STI571 therapy. In the subgroup of patients treated with STI571 at a dose of 300 mg or higher, 53/54 (98%) achieved complete hematological response and 29/54 (54%) achieved overall cytogenetic response, which was complete in 7/54 (13%) of treated patients (see below for response definitions) [30,31].

Three levels of response were reported with TKi-based treatment: (1) hematologic, which was defined as the normalization of peripheral blood counts, without circulating immature elements and no-palpable spleen; (2) cytogenetic, which was defined as complete with no PH+ detectable, partial with PH+ 1–35%, minor with PH+ 36–65%, minimal with PH+ 66–95%, and no response with PH+ >95%; and (3) molecular, which was defined as complete molecular response (CMR) with no BCR-ABL1 transcript detectable, deep molecular response (DMR) with BCR-ABL1 transcript <0.01% (where the molecular response 4 (MR4) is ≤0.01% IS, MR4.5 ≤ 0.0032% IS, and MR5 < 0.001%), major with molecular response (MMR) BCR-ABL1 transcript <0.1% [32].

The IRIS trial was an important randomized phase III trial that compared imatinib to interferon plus cytarabine in a population of 1106 patients with newly diagnosed chronic phase CML. The results at 12 months follow up were excellent in terms of complete cytogenetic remission (CCyR) rate (68% vs. 7%) and BCR-ABL1 transcript levels evaluated in the patients with cytogenetic remission, which represented at least a 1000-fold decrease in 57% vs. 24% patients treated with imatinib vs. interferon plus cytarabine, respectively [33]. After long-term treatment with imatinib, CML clones can become refractory [34]. The 5-year update of the IRIS study showed that among a total of 553 patients [35], 35 cases (6%) progressed to accelerated phase or blast crisis, 14 patients (3%) had hematological relapse, and 28 patients (5%) lost major cytogenetic response. This loss of response was probably due to point mutations in the BCR-ABL1 ATP-binding site, activation loop, or catalytic domain, which prevent imatinib from binding the oncogenic kinase. These mutations were described for the first time in 2001 [36].

The second generation TKi, dasatinib, was designed with a 100-fold increased potency in inhibiting BCR-ABL1 compared to imatinib [37]. In early phase I and II trials, dasatinib showed tolerability and efficacy in previously imatinib-treated patients with CML [38,39]. Efficacy was confirmed in the randomized phase III DASISION study where first-line treatment with dasatinib showed better results compared to imatinib after 12 (CCyR: 83% vs. 72%; MMR: 46% vs. 28%) and 24 months treatment [40,41] (CCyR: 86% vs. 82%; MMR: 64% vs. 46%). Furthermore, the recent 5-year update of the DASASION trial confirmed the long-term efficacy of dasatinib compared to imatinib (5-year-MMR and MR 4.5: 76% and 42% for dasatinib vs. 64% and 33% for imatinib, respectively; *p* = 0.0022 and *p* = 0.0251) [42].

Nilotinib is another second-generation TKi, which is effective against most of BCR-ABL1 point mutations (T315I excluded) [43]. The clinical efficacy of nilotinib was demonstrated in the randomized phase III ENESTnd trial in which different doses of nilotinib were compared to a standard dose of imatinib in newly diagnosed chronic phase CML. Outcomes for both nilotinib arms (300 mg or 400 mg twice daily) were significantly superior to imatinib at 24 months in terms of MMR (71% vs. 67% vs. 44%), complete molecular response (26% vs. 21% vs. 10%), and progression to accelerate or blastic phase [44].

In addition, the superior efficacy of nilotinib vs. imatinib was confirmed by the 5-year (MR 4.5: 54% and 52% for nilotinib 300 mg and 400 mg twice daily vs. 31% for imatinib) [45] and 10-year updates (MR 4.5: 61% for both nilotinib arms vs. 39.2% for imatinib arm) [46] of ENESTnd trial.

The threonine at position 315 of the BCR-ABL1 is important for the interaction of most TKi with BCR-ABL1. In the case of T315I mutation (threonine is replaced by isoleucine), a conformational change in BCR-ABL1 occurs that prevents the hydrogen bond between the TKi and oncogenic kinase required for the TKi to inhibit kinase activity [43]. The spectrum of reported BCR-ABL1 mutations associated to TKi resistance is shown in Table 1 [47].

Ponatinib was the first third-generation TKi specifically designed to overcome resistance from T315I mutation by its inhibitory action through the creation of an ethynyl bond [48]. The clinical efficacy of ponatinib was evaluated in the single-arm phase II PACE trial where 449 heavily pretreated patients with CML or PH+ acute lymphoblastic leukemia (ALL) were enrolled after dasatinib/nilotinib failure or with theT315I mutation. Major CCyR and MMR rates were 56%, 46%, and 34%, respectively with ponatinib treatment. Interestingly, no mutations conferring resistance to ponatinib were detected. However, serious arterial thrombotic events were observed in 9% of patients [49]. The multicenter phase III randomized EPIC trial (ponatinib vs. imatinib in 307 treatment-naive CML) showed significantly higher rates of MMR (41% vs. 18%) and MR4.5, which was defined as BCR-ABL1 ≤ 0.0032% IS [50] (15% vs. 0) at any time for patients treated on the ponatinib arm. However, 7% vs. 0.7% patients experienced severe arterial thrombotic events with ponatinib versus imatinib, leading to the early discontinuation of the trial due to concern for ponatinib toxicity [51].

Bosutinib is a potent (200 times imatinib) dual SRC/ABL kinase inhibitor that was approved for patients with CML with resistance or intolerance to imatinib, following the results of a phase I/II trial [52]. In the randomized phase III study BFORE, 536 patients with newly diagnosed chronic phase CML were enrolled 1:1 to receive bosutinib or imatinib; at 12 months, CCyR (77.2% vs. 66.4%; *p* = 0.0075) and MMR (47.2% vs. 36.9%; *p* = 0.02) rates were significantly higher for patients treated with bosutinib versus imatinib [53].

The ATP-binding site of BCR-ABL1 is the preferred target of kinase inhibitors, and most resistance-associated mutations occur at this site. The Specifically Targeting the ABL Myristoyl Pocket (STAMP) Asciminib (ABL001) was an innovative allosteric inhibitor that targets the BCR-ABL1 myristoyl pocket and was specifically designed to overcome all the mutations in the ATP-binding site [54]. However, rare mutations located in the myristate pocket were described, suggesting that asciminib might lose its efficacy when used in monotherapy; the association between the classic ATP-binding site inhibitors and asciminib might promote simultaneous BCR-ABL1 inhibition with combined treatment, reducing the risk of treatment resistance.

The first-in-human multicenter dose escalation phase I trial of asciminib enrolled 101 patients with CML after ≥2 prior TKi or with PH+ ALL after ≥1 prior TKi treatment, and it identified a recommended dose of asciminib of 40 mg BID for CML patients. Fifty-five patients treated >3 months with asciminib achieved a MMR of 23.6% (13 of 55), 43.2% (16 of 37), and 57.1% (20 of 55) at 3, 6, and 12 months, respectively [55]. The phase II trial ASC4MORE (NCT03578367) is currently ongoing to evaluate the efficacy of asciminib after imatinib in patients with chronic phase CML without deep molecular response (DMR) after ≥12 months of imatinib treatment. Eighty patients were randomized to asciminib 40 mg or 60 mg QD plus imatinib 400 mg QD, continued imatinib 400 mg QD, or switch to nilotinib 300 mg BID. The results of this study are pending [56]. The multicenter phase III ASCEMBL study compared single agent asciminib versus bosutinib in 233 patients with chronic phase CML after failure ≥2 TKi. Treatment with asciminib was associated with significantly improved response compared to bosutinib in terms of 24-week MMR rate (25.5% vs. 13.2%; *p* = 0.029) [57]. The main features of BCR-ABL inhibitors are resumed in Table 2.

**Table 2:** Characteristics of first line BCR-ABL inhibitors.

	N	Clinical Trial	Trial Phase	CML Phase	TKI Dosage	Response	Bcr-AblT315I
Imatinib vs. interferon + low-dose of cytarabine	1106	IRIS (NCT00333840)	III	Chronic	400 mg/die	MCR: 87% CCR: 76%	No
Imatinib vs. historic experience	389		Retrospective [58]	Accelerated	600 mg/die	MCR: 49% CCR: 43%	no
Dasatinib vs. imatinib	519	Dasision (NCT00481247)	III	Chronic	100 mg/die	CCR: 83% MMR: 46%	no
Dasatinib	174	START-A	II	R/R Accelerated	140 mg/die	MCR: 39% CCR: 32%	no
Dasatinib	109 48	START-C	II	R/R myeloid blast R/R lymphoid blast	140 mg/die	MCR: 33% CCR: 23% MCR: 52% CCR: 46%	no
Nilotinib vs. imatinib	846	ENESTnd (NCT00471497)	III	Chronic	600mg/die 800 mg/die	CCR: 80% MMR: 44% CCR: 78% MMR: 43%	no
Nilotinib	136		II [59]	R/R Accelerated	800 mg/die	MCR: 31% CCR: 19%	no
Bosutinib vs. imatinib	536	BFORE (NCT02130557)	III	Chronic	400 mg/die	CCR: 77% MMR: 47%	no
Ponatinib	267 83 62	PACE (NCT01207440)	II	Chronic Accelerated Blastic	45 mg/die	MCR: 56% CCR: 46% MMR: 34% MCR: 39% MCR: 23%	yes

CCR, complete cytogenetic response; MCR, major cytogenetic response; MMR, major molecular response.

### 2.3. TKi Treatment Discontinuation in CML

Until recently, therapy of CML with TKi was not considered curative, and therefore, patients were treated indefinitely until intolerance or disease progression. Many recent trial results demonstrated that a proportion of patients can stop TKi treatment without experiencing a disease relapse and can potentially be cured. Nowadays, TKi treatment discontinuation is one of the most important goals for younger patients diagnosed with CML [60].

The prospective, multicenter, Stop Imatinib (STIM) trial evaluated imatinib discontinuation for 100 patients with CML >18 years old who achieved CMR (defined as >5-log reduction in *BCR–ABL* and ABL levels and undetectable transcripts by quantitative RT-PCR) for at least 2 years, after ≥3 years of TKi treatment. With a median follow up of 17 months, 61% of 69 patients (at least 12 months of follow up) experienced a molecular relapse. However, all patients with molecular relapse responded to imatinib rechallenge (26 achieved a sustained CMR and 16 achieved a decrease in BCR-ABL1 transcript levels) [61]. Interestingly, the recent multicenter phase II trial DASFREE assessed treatment-free remission (TFR) after dasatinib discontinuation in 84 patients with chronic phase CML. After 24 months discontinuation, TFR was 46%, and 44/45 (98%) patients re-achieved MMR (median 2 months; range 1–4) and 43/45 (96%) re-achieved DMR (median 3 months; range 2–18) after dasatinib rechallenge. In multivariable analysis, the duration of dasatinib treatment (>median; *p* = 0.005), line of therapy (first-line; *p* = 0.0138), and age (>65 years; *p* = 0.0012) were associated with 2 year-TRF [62]. In the EURO-SKI trial, the logistic progression model showed that 6 month-TRF is significantly associated with the duration of deep molecular response (DMR, defined as 10,000-fold reduction in BCR-ABL1 transcripts) with TKi, as described in Table 3 [63].

In summary, current guidelines recommend TKi discontinuation for patients who have been in therapy with TKi for at least 3 years and who achieved a DMR of at least 2 years [64,65].

## 3. Chronic Lymphocytic Leukemia

### 3.1. Kinase Pathways in CLL

CLL is a clonal disorder of mature B lymphocytes characterized by the co-expression of CD5, CD19, CD23, and CD20 that accumulate in blood, lymph nodes, spleen, and bone marrow. The median age at diagnosis is 72 years, and most patients are initially observed without treatment [6] The B-cell receptor (BCR) signaling pathway is constitutively activated and fundamental to the pathophysiology of the leukemia cells in CLL [66]. The BCR complex consists of a surface transmembrane immunoglobulin (Ig) receptor associated with Igα (CD79A) and Igβ (CD79B) chains and multiple downstream intracellular signaling proteins [67]. The BCR signaling pathway is essential for B-cell survival and proliferation; the loss of one of the key intracellular downstream signaling proteins, Bruton tyrosine kinase (BTK) leads to Bruton’s X-linked agammaglobulinemia (known as X-linked agammaglobulinemia (XLA)), which is a severe immunodeficiency characterized by the developmental arrest of B-cell precursors and no immunoglobulin production [68,69].

Under physiological conditions, two different signals are propagated by the BCR signaling complex: (1) a basal, constitutive survival signal, which is antigen independent and mediated through PI3K; and (2) the signal triggered by interaction of surface Ig with extracellular antigens that promotes the recruitment of downstream kinases such as LYN kinase, spleen tyrosine kinase (SYK), BTK, and PI3K. In the later signaling, upon binding of antigen and triggering of Ig signaling, LYN and SYK phosphorylate BTK, activating phospholipase Cλ2 (PLCλ2), MAP kinases, AKT, and nuclear factor kappa-light-chain-enhancer of activated B cells (NF-кB) [67]. BTK and PI3K were central to B cell signaling and therefore considered as potential targets for blocking the survival and proliferation of BCR signals with small molecule inhibitors [70,71]. However, CLL cells have different BCR pathways compared to physiological B lymphocytes: ZAP-70 is a protein that, when overexpressed in CLL cells, enhances BCR signaling, acting as an adapter protein rather than its kinase activity [72,73]. In addition, TOSO, named Fas inhibitory molecule 3 (FAIM3), is an IgM Fc receptor (FcμR) that is over-expressed in CLL clonal cells; the role of TOSO is not completely clear yet, but it might interact with SYK, enhancing BCR pathways and promoting apoptosis arrest [74].

**Figure 2:**
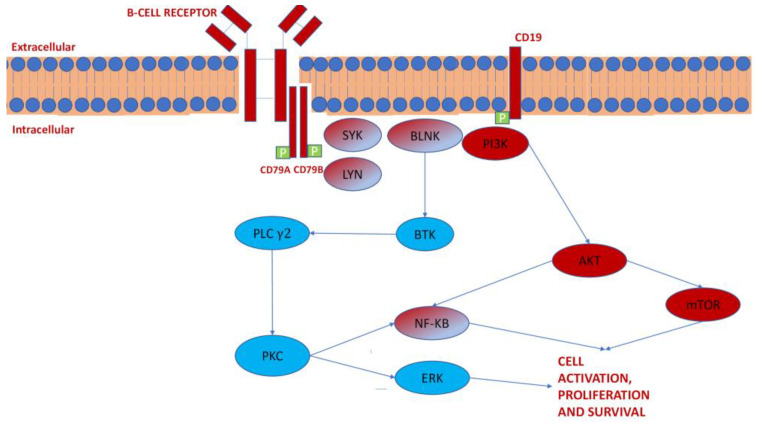
B-cell receptor signaling pathway.

A summary of BCR pathways is shown in Figure 2.

Surface B-cell receptor and down-stream signaling proteins participate in pro-survival, proliferation, migration, and apoptosis signals to the CLL cells.

Blue indicates the BTK-dependent pathway; red is the PI3K-dependent pathway; red and blue indicate common proteins.

### 3.2. TK Inhibition in CLL

Historically, first-line treatment for CLL was based on chemoimmunotherapy (CIT) consisting of CD20 monoclonal antibody (mAb), rituximab [75], or obinutuzumab [76], combined with different chemotherapeutic agents such as chlorambucil [77], bendamustine [78], or fludarabine and cyclophosphamide [79]. However, based on multiple phase III clinical trials, BTK-inhibitor-based treatment has proven superior to CIT in terms of efficacy and safety. Two different families of TKi are currently approved: BTK inhibitors (BTKi) [80] (in first-line and relapsed/refractory CLL) and PI3K inhibitors (PI3Ki) (in relapsed/refractory CLL) [81].

The mechanism of BTK inhibition may be irreversible or reversible; the currently available inhibitors are irreversible. Irreversible BTKi inactivate BTK by blocking the binding of ATP by a very stable covalent bond with the sulfhydryl group of Cys481, which is located in the BTK ATP-binding site. In contrast, reversible BTK inhibition is based on the stronger non-covalent binding of BTK, which blocks ATP binding and does not require Cys481 [82].

The family of irreversible BTKi consists of seven molecules (ibrutinib, acalabrutinib, zanubrutinib, tirabrutinib, spebrutinib, remibrutinib, and evobrutinib). Ibrutinib was approved by the FDA in 2014 for relapsed/refractory (R/R) CLL following the results of a single-arm trial in which 48 R/R patients with CLL achieved a high overall response rate (ORR; 58.3%) and response duration (DOR; 5.6–24.2 months) [83]. The indication was expanded to include R/R del(17p) or mutated-*TP53* CLL based on the RESONATE-17 trial [84,85], demonstrating a high ORR (64%) and 24-months PFS (63%) in 144 patients with R/R del(17p) CLL. Interestingly, patients with del(17p) or complex karyotype R/R CLL have shorter PFS also with ibrutinib treatment compared to patients without these genetic abnormalities [86,87].

Ibrutinib was approved as a first-line treatment of CLL based on the phase III RESONATE-2 trial [88], which demonstrated significantly improved PFS (median, not reached vs. 18.9 months; *p* < 0.001) and OS (24-month OS rate, 98% vs. 85%; *p* = 0.001) for ibrutinib compared to chlorambucil in 269 treatment-naïve (TN) patients with CLL more than 65 years old.

Other phase III trials in first-line treatment of CLL, including LLUMINATE and E1912, evaluated ibrutinib with or without CD20 mAb versus CIT. Specifically, treatment with ibrutinib plus obinutuzumab or ibrutinib plus rituximab resulted in significantly improved median PFS compared to treatment with chlorambucil–obinutuzumab in iLLUMINATE or fludarabine–cyclophosphamide–rituximab in the E1912 trial, respectively [89,90]. Additionally, the three-arm ALLIANCE trial evaluated ibrutinib with our without rituximab versus bendamustine plus rituximab in ≥65 years patients with treatment-naïve CLL. Both the ibrutinib arms showed superior PFS with respect to bendamustine–rituximab [91].

The 6-year update of the phase III RESONATE trial showed significantly longer median PFS (44.1 vs. 8.1 months; *p* < 0.001) for ibrutinib vs. ofatumumab treatment in patients with R/R CLL [92]. Furthermore, treatment with combined ibrutinib, rituximab, and bendamustine improved median PFS compared to bendamustine plus rituximab in patients with R/R CLL in the randomized double-blind phase III HELIOS study [93] A recent update of the HELIOS trial reported a median follow up of 34. 8 months and longer median PFS (not reached for ibrutinib plus bendamustine–rituximab vs. 14.3 months for bendamustine–rituximab) and higher 36-month PFS (68% vs. 13.9%, respectively) [94]. However, the value of bendamustine plus rituximab when combined with continuous ibrutinib in this regimen is debated. Continuous and indefinite treatment with ibrutinib is associated with side effects, usually mild (grade I–II), which need to be managed. For example, skin manifestations are common (up to 27%) and consist of cutaneous rash, ecchymosis, and petechiae [86]. An increased risk of bleeding due to ibrutinib-induced platelet dysfunction is another long-term side effect [95]. In clinical trials, the rate of major bleedings (grade ≥ 3), including subdural hematomas gastrointestinal and intracerebral bleedings, is reported in up to 8–10% of patients; however, this risk might be influenced by concomitant treatments with anticoagulant or antiplatelet agents [96,97]. Cardiac side effects, including atrial fibrillation (AF), hypertension, and ventricular arrhythmias are also reported during ibrutinib therapy. Notably, AF is the most frequent reason for ibrutinib discontinuation [98]. The incidence of AF increases with longer treatment period (3–7% < 18 months; 9–16% ≥ 18 months) [97].

Acalabrutinib is a second generation, more specific and selective irreversible BTKi evaluated as single agent (*n* = 179) or combined with obinutuzumab (*n* = 179) versus chlorambucil plus obinutuzumab (*n* = 177) in treatment-naïve patients CLL in the multicenter randomized phase III ELEVATE-TN trial. This trial demonstrated significantly improved PFS for both acalabrutinib-containing arms over chlorambucil plus obinutuzumab. Acalabrutinib was FDA approved in November 2019 as a first-line treatment for CLL [99]. The multicenter randomized phase III ASCEND trial also demonstrated improved PFS for acalabrutinib over the investigator’s choice (idelalisib–rituximab or bendamustine–rituximab) in patients with R/R CLL [100].

An ongoing randomized, multicenter phase III non-inferiority study to evaluate ibrutinib vs. acalabrutinib for treatment of patients with high-risk cytogenetic R/R CLL (ELEVATE-RR; NCT02477696) [del(17p) or del(11q)] reported improved tolerability and toxicity for acalabrutinib with reduced incidence of all grade atrial fibrillation and non-inferior efficacy for acalabrutinib versus ibrutinib and similar efficacy [101]. In addition, the ALPINE trial (NCT03734016) is a multicenter phase III trial currently evaluating zanubrutinib (irreversible BTKi) versus ibrutinib also preliminarily reporting reduced risk for all-grade atrial fibrillation for zanubrutinib over ibrutinib and with a trend for improved PFS with zanubrutinib [102].

Tirabrutinib [103], spebrutinib (NCT01732861-NCT01975610), remibrutinib [104], and evobrutinib (NCT02975349) are being evaluated in clinical trials in the setting of B-cell malignancies, including CLL, and in autoimmune diseases.

**Figure 3:**
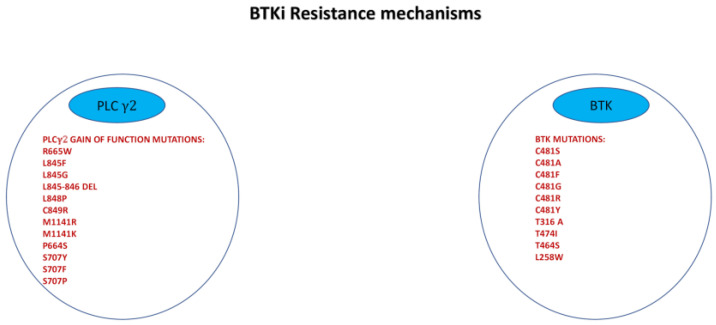
Spectrum of mutations related to BTKi resistance.

Despite very good responses and highly durable disease control achieved with BTK inhibition, some patients might develop refractory disease to irreversible BTKi and progress during treatment. Notably, resistance to irreversible BTKi has been associated with substitution of the cysteine in position 481 with a serine (C481S mutation) as the most frequent mechanism of CLL cell resistance. The cysteine residue is essential for the creation of the irreversible covalent bound between BTK and irreversible inhibitors [105]. Other mutations involving C481 have been described but are less common, including C481F, C481G, C481R, C481Y, and C481T [106,107]. Another mechanism of resistance to irreversible BTKi, although less common and BTK mutation, is the acquisition of gain of function mutations in PLC γ2 protein (R665W and L845F), leading to autonomous BCR activity [108]. Subsequently, other mutations have been discovered and reported in Figure 3 [109].

The reversible BTKi (pirtobrutinib, vecabrutinib, ARQ531, fenebrutinib), potentially overcomes C481 mutations by very strong non-covalent interactions (hydrogen, ionic bonds, and hydrophobic interactions) with BTK that block ATP binding and do not require C481 [82]. These other reversible BTKi showed good inhibitory activity in vitro against wild-type and mutated BTK. ARQ531 and fenebrutinib showed good results in early clinical evaluations in B-cell malignancies, including CLL. In contrast, the clinical development of vecabrutinib was discontinued because of no clinical activity in CLL [110,111,112,113,114]. Clinical trials of reversible BTKi are in early stages. Pirtobrutinib [115] is a reversible inhibitor being evaluated in a phase I/II study in patients with B-cell malignancies, including CLL, showing 62% ORR in the R/R CLL subgroup (*n* = 121) at the recommended dose of 200 mg/day.

Idelalisib was the first PI3Kδ-inhibitor (PI3Kδi) developed for the treatment of patients with CLL. Early phase I and II clinical trials demonstrated efficacy in terms of ORR and median PFS, showing durable responses [116,117]. However, treatment-related side effects and toxicities have been challenging with idelalisib. A high rate of treatment-related transaminitis was observed at a median of 28 days on treatment. Hepatic biopsies documented an autoimmune etiopathogenesis [118]. Lymphocytic infiltration was also seen with colitis and pneumonitis reported as treatment-related toxicities associated with idelalisib and have made use of this agent potentially challenging. Two randomized phase III trials confirmed idelalisib efficacy in CLL when combined with bendamustine–rituximab or ofatumumab, but also in these trials, excessive infection (bacterial pneumonia, pneumocystis Jirovecii pneumonia, CMV reactivation) and liver toxicity were observed [119,120]. Idelalisib is currently only approved for patients with R/R CLL, not in first-line treatment.

Duvelisib is a more recently developed PI3Kδi, which demonstrated strong antiproliferative effects in CLL cells [121]. A phase I trial, in which patients affected by lymphoproliferative malignancies were enrolled, showed good results for duvelisib in terms of ORR. The safety profile was similar to idelalisib [122]. In the multicenter, randomized phase III DUO trial, patients with R/R CLL received duvelisib single-agent versus ofatumumab. This trial demonstrated significantly increased ORR and longer median PFS with duvelisib treatment, including in patients with high-risk cytogenetic CLL [123].

Other PI3Ki are in development. Parsaclisib is a next-generation, potent, and highly selective PI3Kδi that has been recently evaluated alone or associated in R/R B-cell malignancies, including CLL, in a phase I/II trial. Parsaclisib showed promising antitumor activity [124]. Umbralisib is an innovative PI3Kδ/CK1ε inhibitor that showed safety and early efficacy in 51 R/R CLL patients in a phase II trial [125].

In contrast to BTKi, resistance mechanisms to PI3Kδi are still poorly understood. A preclinical investigation reported increased or persistent activity of the ERK pathway, which might be involved in PI3Kδi resistance [126]. Furthermore, in mantle cell lymphoma, it was shown in vitro that a higher PIK3CA (encoding for p110α)/PIK3CD (encoding for p110δ) ratio of messenger RNA transcripts is associated to PI3Kδi resistance [127]. In addition, MYC amplification was observed as a potential resistance mechanism to PI3Ki in breast cancer models [128].

## 4. Discussion

The development and availability of targeted therapies based on tyrosine kinase inhibition has fundamentally changed the management of CML and CLL. A greater selectivity toward neoplastic cells, higher efficacy rates, oral administration, and safer toxicity profile made this therapeutic approach preferable over conventional chemotherapy. Furthermore, high cytogenetic risk diseases such as CLL with del(17p) are much more effectively treated with tyrosine kinase inhibitors.

In recent years, especially in younger patients, achieving undetectable MRD (defined as the persistence of a very low number of neoplastic cells after or during the treatment, detectable by molecular or flow cytometry analysis) [129,130] has become a very important and realistic goal with TKi-based treatment, considering the desire for treatment discontinuation. Several trials in patients with CML have already shown that in predetermined conditions (CMR for at least 2 years after ≥3 years of TKi treatment), the BCR-ABL1 inhibitor can be discontinued and rechallenged in case of molecular relapse. Conversely, TKi discontinuation is not yet recommended in CLL, since the remissions are not deep with TKi-based treatment (extremely rare MRD remissions), and a worse outcome was described in patients who underwent ibrutinib interruption for adverse effects [131]. Further clinical trials and follow up are recommended to identify the risk conditions for indefinite therapy in patients with CLL.

In conclusion, we highlight CML and CLL as examples of hematological malignancies in which the TKi-based approach was transformative and has remarkably improved outcomes for patients with these diseases. The discovery of new intracellular kinase pathways that may be inhibited and the approval of further tyrosine kinase inhibitors is anticipated in the future because of the intrinsic genomic instability of hematological neoplastic cells, which may develop resistance to available therapies.

## Figures and Tables

**Table 1:** Spectrum of BCR-ABL1 mutations, their localization, and their relationship with TKi.

TKI	Strong Resistance	Mild–Moderate Resistance
Imatinib	Y253–E255–T315	M244–L248–G250–Q252–F317–M351–M355–F359–H396
Dasatinib	T315	V299–F317
Nilotinib	T315	L248–Y253–E255–F359
Bosutinib	T315–V299	L248–G250–E255–F317
Ponatinib		T315–E255
Asciminib	A337–W464–P465–V468–I502	

P-loop mutations: M244, G250, Q252, Y253, and E255; gatekeeper residue (T315 and F317); SH2 contact and C-lobe (M351, F359); activation loop (H396).

**Table 3:** Evaluation of the variables associated to 6-month major molecular response after TKI discontinuation; data from EURO-SKI trial (*N* = 448).

Parameter	Odds Ratio (95%CI)	*p* Value
Age at stop of TKI (years)	1.9 (0.95–1.26)	0.21
Interferon pretreatment	2.50 (1.43–4.36)	0.0013
Duration of interferon pretreatment (years)	1.38 (1.12–1.69)	0.0022
Duration of TKI treatment (years)	1.16 (1.08–1.25)	<0.0001
DMR duration while receiving TKI (years)	1.16 (1.08–1.25)	0.00011
Time of TKI treatment before DMR (years)	1.02 (0.93–1.13)	0.66
